# Toxicological landscape of Fuzi: a comprehensive study on the spatial distribution of toxicants and regional neurotoxicity variability in zebrafish

**DOI:** 10.3389/fphar.2024.1500527

**Published:** 2025-02-05

**Authors:** Xiaoqi Pan, Tianyu Liang, Han Feng, Weiying Liu, Qiaoxin Mou, Xiaoyu Yan

**Affiliations:** ^1^ State Key Laboratory of Southwestern Chinese Medicine Resources, Chengdu University of Traditional Chinese Medicine, Chengdu, Sichuan, China; ^2^ School of Public Health, Chengdu University of Traditional Chinese Medicine, Chengdu, China; ^3^ School of Pharmacy, Chengdu University of Traditional Chinese Medicine, Chengdu, China

**Keywords:** Fuzi, diester-type alkaloids, regional differences neurotoxicity, MALDI-MSI, RNA sequencing

## Abstract

Fuzi, a Chinese herb widely used in clinical settings, exhibits varying levels of toxicity depending on its geographical origin. Diester-type alkaloids are the primary contributors to the toxicity of Fuzi. This study aims to investigate regional differences and underlying mechanisms of Fuzi-induced neurotoxicity across China. Matrix-assisted laser desorption/Ionization mass spectrometry imaging (MALDI-MSI) method was employed to map the spatial distribution of six key diester-type alkaloids from Fuzi samples originating from five major regions. The results showed that the diester-type alkaloids were primarily distributed in the cuticle of Anguo- and Ludian-Fuzi, in the cuticle, cork, and pith of Butuo-Fuzi, in the phloem and pith tissues of Chenggu-Fuzi, and in the cuticle, cork, inner phloem, and pith of Jiangyou-Fuzi. When zebrafish were exposed to a Fuzi decoction for 24 h, it was observed that Jiangyou-Fuzi induced the most significant neurobehavioral abnormalities, lipid peroxidation damage, and aberrant neurotransmitters release. RNA sequencing analysis further indicated that the amino acid metabolism, ErbB, cGMP-PKG, and p53 signaling pathways-regulated by changes in the expression of Glub, Mao, GAB1, PRKG1B, PSEN2, and BAXα genes were disrupted to varying extents by Fuzi from different origins. In summary, the regional variability in the neurotoxicity of Fuzi can be attributed to differences in the distribution of its active compounds and underlying mechanisms. Among the samples tested, Jiangyou-Fuzi exhibited the highest neurotoxicity, followed by Anguo-, Chenggu-, Ludian-, and Butuo-Fuzi.

## 1 Introduction

Fuzi, derived from the *Aconitum* species, is one of the most crucial herbal medicines in traditional Chinese medicine. Renowned for its potent therapeutic properties and a broad spectrum of biological and pharmacological effects, Fuzi has been indispensable in healthcare throughout Chinese medical history (Liu et al., 2021; Tian et al., 2023). It has been commonly used to treat a variety of diseases, including rheumatoid arthritis, cardiovascular diseases, tumors, skin wounds, depression, diarrhea, gastroenteritis, and edema (Gao et al., 2010; Do et al., 2019; Wang et al., 2023). However, it is crucial to note that Fuzi exhibits considerable toxicity in both scientific research and clinical application, primarily affecting the nervous, cardiovascular, and digestive systems (Shi et al., 2023). This toxicity can be attributed to factors such as excessive dosage, prolonged treatment duration, improper processing, or inappropriate combinations (Hao et al., 2020).

The cultivation of Traditional Chinese Medicine (TCM) herbs across multiple regions is a prevalent phenomenon, significantly impacting the quality of these herbal remedies. Fuzi, for instance, is grown in various provinces throughout China, including Sichuan, Shanxi, Guizhou, Hunan, Hubei, Gansu, and Yunnan. The efficacy and potential toxicity of Fuzi varies widely depending on its geographic origin. The majority of studies have focused on the cardiac toxicity of Fuzi, yet the underlying mechanism of its neurotoxicity remains underexplored and requires further investigation. Notably, there is a dearth of comparative studies examining the neurotoxic effects of Fuzi sourced from different regions. To date, numerous chemical components have been isolated and identified within Fuzi. Among these, *Aconitum* alkaloids have been proven to be key active and toxic components. Specifically, diester-type alkaloids constitute the most abundant, potent, and toxic components within Fuzi. Notably, aconitine, mesaconitine, and hypaconitine belong to this category and have all been demonstrated the ability to elicit severe arrhythmias in zebrafish, with lethality observed 24 h post-exposure to a concentration of 250 µM (Fang, 2018). Additionally, Our previous study has demonstrated that aconitine induces nerve damage by disrupting dopamine homeostasis and activating the AC/cAMP/PKA pathway in zebrafish (Zhou et al., 2019).

Matrix-assisted laser desorption/ionization mass spectrometry imaging (MALDI-MSI) is a cutting-edge tissue imaging technique, offering a label-free approach for localizing various metabolites within tissue sections (Bhandari et al., 2015; Boughton et al., 2016; Sun et al., 2019). Researchers have successfully used MALDI-MSI to map the spatial distribution and dynamics of metabolites in diverse plant tissues, providing insights into the intricate metabolic pathways of plant secondary metabolites (Li et al., 2013; Li B. et al., 2018). However, there have been no reports utilizing MALDI-MSI to investigate the spatial distribution of diester-type alkaloids in Fuzi. More importantly, accurately characterizing diester-type alkaloids in different botanical parts of Fuzi is crucial for understanding the neurotoxic effects of Fuzi from various geographical origins. Additionally, the rapid advancements in high-throughput genomics and bioinformatics technologies in recent years has provided robust, multifaceted, and networked research tools for studying the neurotoxicity mechanisms of Fuzi. Gaining a clear understanding of the neurotoxic mechanisms of Fuzi from different regions is essential for evaluating its safety and quality. Researching Fuzi neurotoxicity through global transcriptome sequencing (RNA-Seq) can directly yield information about toxic-related functional genes and provide more specific pathway details related to neurotoxicity.

In this study, we aimed to compare the neurotoxic effects of Jiangyou-, Chenggu-, Anguo-, Ludian-, and Butuo-Fuzi decoction in zebrafish larvaes, and clarify the precise localization of diester-type alkaloids within Fuzi. Which provide a scientific basis for quality evaluation and clinical application of Fuzi. The spatial distribution of diester-type alkaloids in Fuzi was investigated using MALDI-MSI to compare these alkaloids in microregion. Subsequently, the neurotoxic effects of Fuzi decoction (aqueous extracts) from different production areas on zebrafish larvae were investigated. Additionally, RNA-Seq method was used to identify key genes and targets in the larvae, providing new insights into the neurotoxic mechanisms of Fuzi decoctions.

## 2 Materials and methods

### 2.1 Herbal materials and preparation of samples

Fuzi, which grows in five different producing areas, Ludian, Anguo, Chenggu, Butuo, and Jiangyou, was harvested locally and identified by Gao Jihai, Professor of the State Key Laboratory of Southwest Characteristic TCM Resources. Fuzi was prepared into decoction as follows: boiled 100 g Ludian-Fuzi in 1,000 mL pure water for 1 h, repeated three times and then concentrated the three water decoctions into an extract. The water extract of Fuzi from other producing areas was prepared as above. The ratios of crude drugs in the decoction of Fuzi in each production area were 0.986842, 0.862069, 0.961538, 0.833333, and 0.877193, respectively.

### 2.2 MALDI-MSI

Fuzi was immersed in water for 5 h, and was then flash-frozen at −80°C. The frozen sample pieces were axially fixed on a Cryostat Microtome (Thermo Fisher Scientific CryoStar NX50, America) by water and sectioned into 30 µm slices at −20°C. The slices were thaw-mounted on indium-tin oxide (ITO) coated glass slides (Bruker Daltonics, Germany) for the optical imaging and application of the matrix. The optical image of the tissue section was captured on an Umax Powerlook III scanner (Umax Technologies, Fremont, California, United States) with a resolution of 1,200 dpi. MALDI matrices were coated using an ImagePrep electronic matrix sprayer (HTX Imaging Sprayer, American). 2,5-Dihydroxybenzoic acid (DHB) was dissolved in acetonitrile: water (50: 50, v/v) at a concentration of 25 mg/mL as MALDI matrix. The matrix spraying parameters were carefully optimized referring to the method provided by the instrument. An MRMS-MALDI analyzer (Bruker Daltonics, Bremen, Germany) was equipped with a 2,000 Hz, 355 nm Nd: YAG laser (minimum spot diameter at 20 µm) for MSI. The sample surface was irradiated with laser shots in the positive mode of the mass spectrometer. Then the surface of the sample was scanned with a spatial resolution of 100 µm. Ions in the mass range of m/z 100–1,000 were detected. The MS images were viewed and processed by using FlexImaging 5.0 software (Bruker Daltonics, Germany) and Zhou et al. (2019). Software (GmbH, Bremen, Germany).

### 2.3 Zebrafish strains, oviposition, and maintenance

Wild-type AB line zebrafish were purchased from Nanjing YiShuLiHua Biotechnology Co., Ltd. (Nanjing, China). All fish experiments were performed following the guidelines issued by the animal ethics committee (AAALAC Certiffcate No. 001458). The zebrafish were raised in the fish house with a daily light/dark cycle of 14 h/10 h, respectively. The water temperature of the aquaculture system was about 28°C, pH 7.2–7.6, conductivity 500–550 μs/cm, and water hardness 53.7–71.6 mg/L CaCO_3_. Feed freshly hatched brine shrimp regularly twice a day. Six dpf zebrafish larvae were collected as experimental subjects by being exposed to different production areas of Fuzi decoction.

### 2.4 Acute toxicity assay

Fuzi decoction was diluted with fish-raising water to different concentrations in each group. Larvae at 6 dpf were placed in a 24-well plate with a density of 10 embryos per well. They were exposed to various concentrations of Jiangyou-Fuzi decoction (1, 2, 3, 4, 5, 7.5, 10, or 20 mg/mL), Anguo-Fuzi decoction (2, 3.5, 5, 7.5, 10, or 20 mg/mL), Chenggu-Fuzi decoction (1, 3, 5, 7.5, 10, or 20 mg/mL), Ludian-Fuzi decoction (1, 3, 5, 7.5, 10, 15, or 20 mg/mL), and Butuo-Fuzi decoction (1, 3, 5, 7.5, 10, 12.5, or 20 mg/mL) for 24 h, respectively. Three parallel samples were set for each concentration following similar criteria with the vehicle controls. The fish with no visible movement (such as the flapping of gills, no response to touching the tail) was dead, and the dead larvae in each group were collected to calculate the median lethal dose (LD_50_) value of Fuzi decoction on zebrafish larvae. The concentration was determined by considering the mortality of larvae after administration of Fuzi decoction with different concentrations in each production area.

### 2.5 Morphological observation

According to the LC_50_ of Fuzi decoction from different regions, the larvae were treated with 2 mg/mL Jiangyou-Fuzi decoction or 3 mg/mL Anguo-, Ludian-, Butuo-, and Chenggu-Fuzi decoction in morphological observation and the following experiments. Potential teratogenicity of Fuzi from diverse areas was estimated by monitoring the morphological defects of larvae at 6 dpf under a stereomicroscope. Morphological indices and body length (μm) were evaluated using ImageJ.

### 2.6 Locomotor behavioral test

Zebrafish at 6 dpf were transferred into 96-well plates (one fish/well) and treated with 2 or 3 mg/mL of Fuzi decoction from different areas per well for 24 h. Then, the swimming behavior of the larvae was monitored by the automated video tracking system (Noldus Co., Netherlands). After 5 min of habituation without light, the behavioral trajectories of larvae for 10 min were captured and used the Ethovision XT12 software (Noldus Co., Netherlands) to track the swimming activity at a rate of 15 positions per second. Total distance traveled and movement speed were obtained through the Ethovision XT12 software.

### 2.7 Measurement of acetylcholine (Ach), glutamic acid (Glu), and γ-aminobutyric acid (GABA)

Zebrafish larvae were euthanized in 0.9% tricaine after being treated with 2 mg/mL or 3 mg/mL Fuzi decoction from diverse areas for 24 h. Then, the Ach, Glu, and GABA levels were measured by ELISA assay kit (Enzymelinked Biotechnology Co., Ltd., China) following the manufacturer’s instructions. All results were normalized to the corresponding total protein content.

### 2.8 Assessment of glutathione (GSH) and malondialdehyde (MDA)

Zebrafish larvae at 6 dpf were exposed to different Fuzi decoctions (2 mg/mL or 3 mg/mL) for 24 h. The 6 dpf of zebrafish were anesthetized with tetracaine and homogenized after euthanasia on ice. GSH and MDA were analyzed simultaneously according to the manufacturer’s instructions. Each experiment contained 20 larvae, and each measured index was repeated in triplicate.

### 2.9 RNA-seq

RNA-seq quantification was performed to investigate the changes in zebrafish larvae mRNA profiles among the different production areas of Fuzi treatments. Isolated RNA was sent to BGI Co., LTD (Wuhan, China) for RNA-seq on the BGISEQ platform. Qualified RNA samples were selected and sequencing results were analyzed with Dr. Tom 2.0 online software (BGI Co., LTD) to identify the differential expression genes (DEGs) (Xu et al., 2020). These were subjected to the Kyoto Encyclopedia of Genes and Genomes (KEGG) pathway enrichment analysis.

### 2.10 Quantitative PCR (RT-qPCR) assay

Total RNA was extracted using the Animal Total RNA isolation kit (Foregene Biotechnology Co., Ltd., China) with a spin column according to the manufacturer’s protocol. Isolated RNA was reverse-transcribed into cDNA using the Master Premix for first-stand cDNA synthesis for RT-qPCR following the standard protocol. The RT-qPCR assay was conducted using Real-time PCR EasyTM -SYBR Green I with the Applied Biosystems 7500 Real-Time PCR System (Analytik jena, Germany). The amplification parameters were 95°C for 3 min, followed by 40 cycles of 95°C for 5 s and 60°C for 20 s. Each sample was analyzed in triplicate, and the relative expression of mRNA was calculated after normalization to β-actin. All primer sequences used are listed in Table 1.

**TABLE 1:** Sequences for RT-qPCR.

Genes	Forward sequences (5′-3)	Reverse sequences (3–5′)
*Glulb*	CTT​CTC​GGC​ACA​GAT​GGT​CA	CCA​ACT​TGG​AAC​TCC​CAC​TGT
*Mao*	TGA​GGT​TTC​GGC​TTT​GTG​GT	AAA​CTT​GCG​CTC​CTG​TCC​TC
*Gab1*	AGC​TGG​GAT​CAT​CCT​CCA​GT	GTC​GCC​ATG​AGC​CCT​GTT​AG
*PRKG1B*	CCT​CGT​CAT​CGA​CAG​AGA​ATC​C	GGG​GAG​GGG​GAA​TGA​AAC​AC
*PSEN2*	CTG​CCA​TGG​TCT​GGA​TGG​TT	GCT​TCA​CTC​CTC​GGT​CTT​CC
*BAXα*	TGT​TTG​CAG​CAG​ATC​GGA​GA	ACC​CTG​GTT​GAA​ATA​GCC​TTG​AT
*β-Actin*	ACC​ACG​GCC​GAA​AGA​GAA​AT	ATG​TCC​ACG​TCG​CAC​TTC​AT

### 2.11 Statistical analysis

In this study, the normality of data was assessed using the Kolmogorov Smirnov test. The homogeneity of variance was evaluated by Levene’s test. The differences between groups were analyzed using one-way analysis of variance (ANOVA) with SPSS 23.0 statistical software, followed by a Tukey post-hoc test. All data are reported as mean ± standard error of the mean (SEM) from at least three independent experiments. *P* < 0.05 was considered statistically significant.

## 3 Results

### 3.1 Spatial distribution of key toxic components of Fuzi from different geographical origins

**FIGURE 1:**
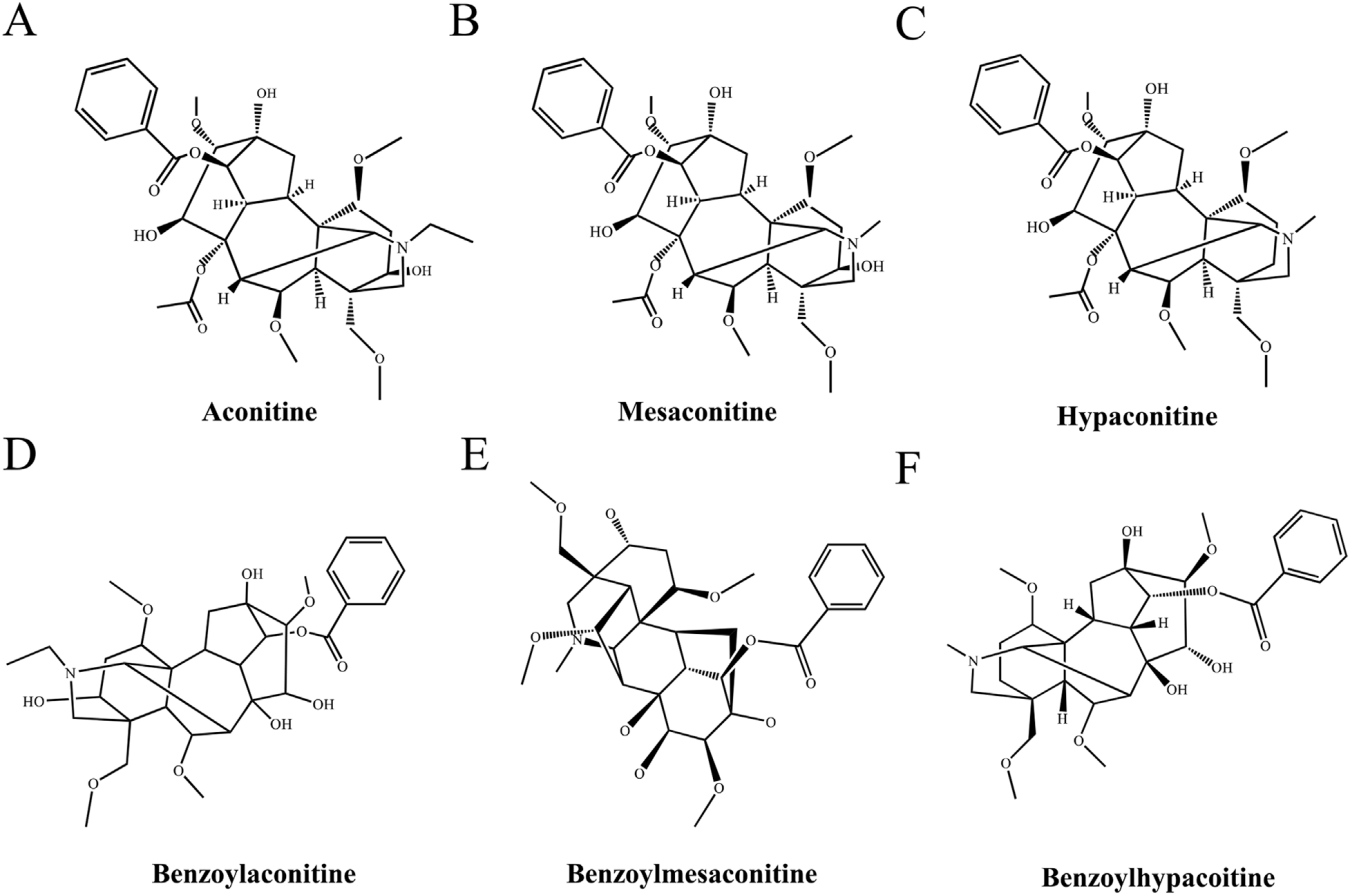
Chemical structures of the diester-type alkaloids in Fuzi. **(A)** Aconitine, **(B)** Mesaconitine, **(C)** Hypaconitine, **(D)** Hypaconitine, **(E)** Hypaconitine, **(F)** Benzoylhypacoitine.

**FIGURE 2:**
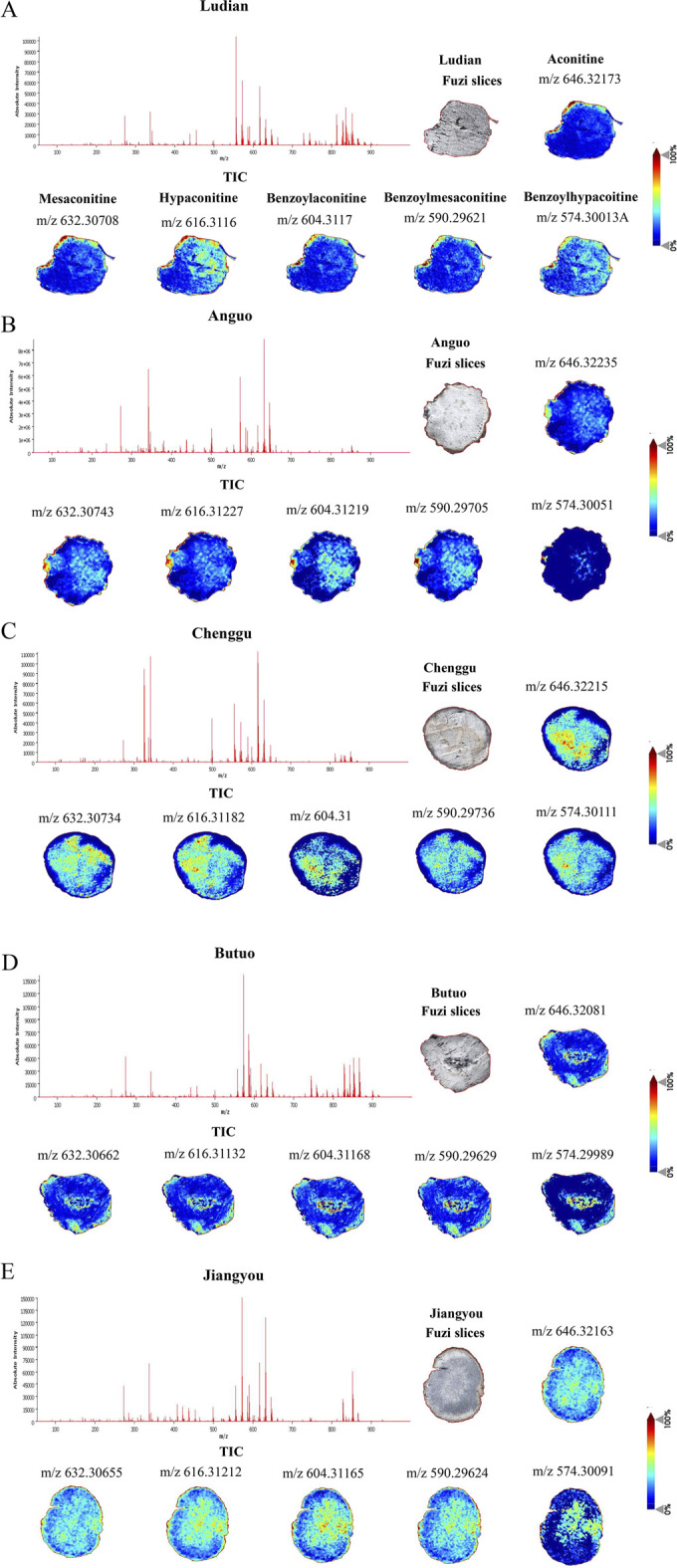
The TIC and spatial localizations of main diester-type alkaloids in Fuzi from different regions by MALDI-MSI (the positive ion mode), scale bar = 4 mm. **(A)** Ludian, **(B)** Anguo, **(C)** Chenggu, **(D)** Butuo, **(E)** Jiangyou regions. Brighter (redder) colors indicate more relative content and darker (bluer) colors indicate less relative content.

To determine the spatial distribution of aconitine, mesaconitine, hypoaconitine, benzoylaconitine, benzoylmesaconitine, and benzoylhypoaconitine in Fuzi slices from different production areas (Figure 1), MALDI-MSI was used to image. Only intact transverse slices of Fuzi were selected for analysis. First, we acquired the total ion chromatogram (TIC) of the Fuzi section from five regions in the positive ion mode. As shown in Figure 2, Fuzi from five different production areas had largely similar TIC. Abundant signal peaks were observed, with high signal abundance in the m/z range of 550–700, and many metabolites in the m/z range of 300–400 and 800–900, while the abundance was relatively low. Coincidentally, all six diesters-alkaloids in Fuzi had m/z value within the range of 500–600, indicating that they were the main toxic components of Fuzi, which was consistent with the results of Xiong et al. (2017).

In plant structure, Fuzi section was further divided into different micro-zones, including cuticular layer, cork, phloem, xylem, and medulla tissues. The spatial distribution characteristics of diester-type alkaloids in different micro-zones of Fuzi sections from five regions were observed. The main toxic substances of Fuzi, aconitine, mesaconitine, hypaconitine, benzoylaconitine, and benzoylmesaconitine, showed stronger ion signals in the cuticular layer, cork, inner xylem, and medulla of Jiangyou-Fuzi slices (Figure 2E). However, the ion signals of Benzoylhypacoitine in the cuticular layer, and cork were relatively weak. The six diester-type alkaloids were found to be mainly distributed in the cuticular layer region of Ludian-, and Anguo-Fuzi (Figures 2A, B). In addition to being mainly distributed in the cuticular layer, and cork parts of Butuo-Fuzi, the six diester-type alkaloids also had strong ion signals in the medulla (Figure 2D). However, the distribution of the 6 diester-type alkaloids in Chenggu-Fuzi was contrary to that in Aanguo- and Ludian-Fuzi, which were mainly distributed in the xylem and medulla tissues (Figure 2C). In short, the relative abundances of diester-type alkaloid in Jiangyou- and Chenggu-Fuzi slices were significantly higher than that of the other regions, and we speculated that the neurotoxic effects of Jiangyou- and Chenggu-Fuzi were stronger than those of other producing areas.

### 3.2 Fuzi decoctions from different origins induced acute toxicity in zebrafish

**FIGURE 3:**
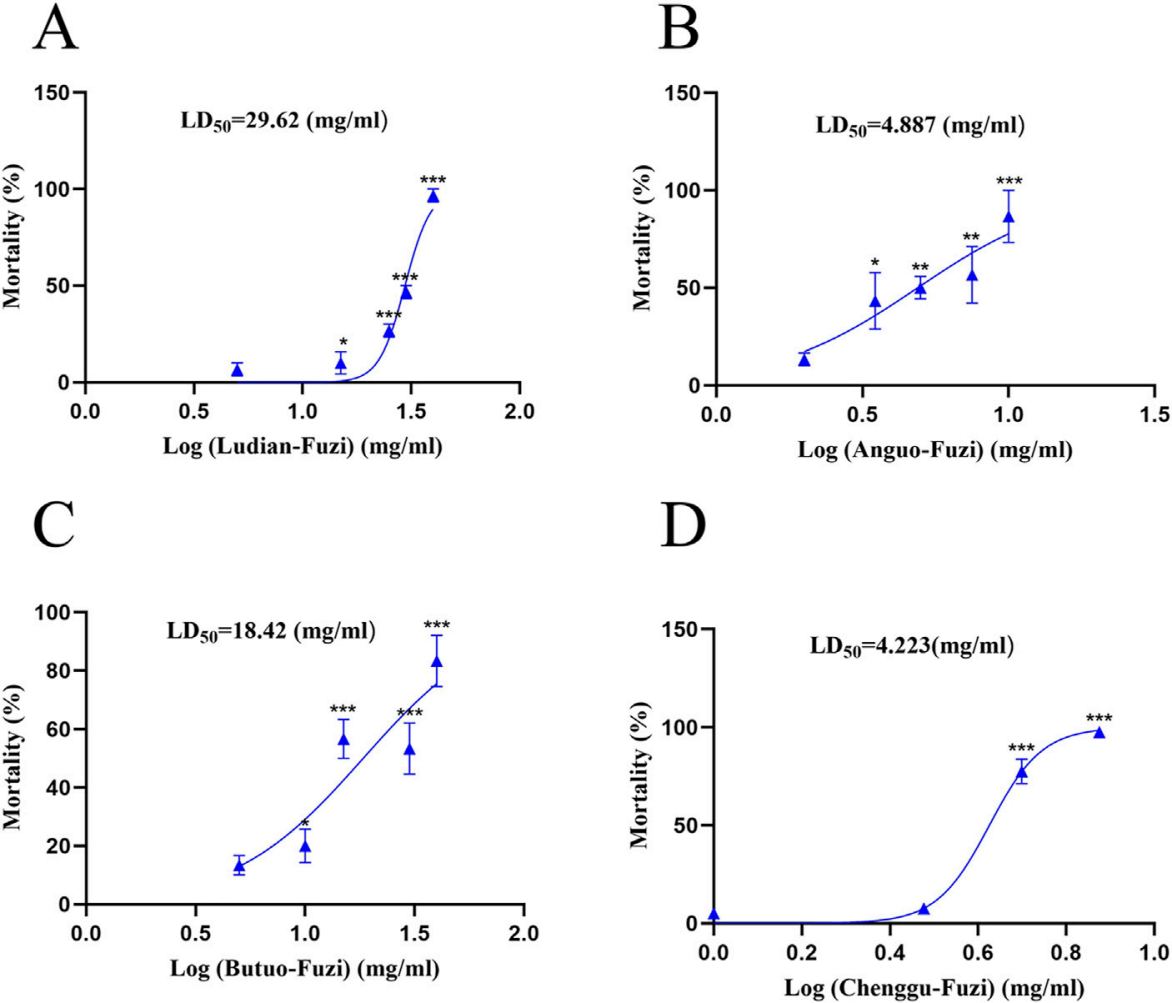
Fuzi decoctions from diverse regions induced acute toxicity in zebrafish larvae. Mortality of zebrafish larvae exposed to Fuzi decoction from Ludian **(A)**, Anguo **(B)**, Chenggu **(C)**, and Butuo regions **(D)**. The values are expressed as mean ± SEM, n = 25. *p < 0.05, **p < 0.01, ***p < 0.001 vs. the control group.

The results of the acute toxicity test showed that zebrafish larvae presented obvious mortality in a concentration-dependent manner after treatment with Fuzi decoction from different regions (Figure 3). Fuzi decoctions from Ludian, Anguo, Chenggu, and Butuo were found to cause 50% zebrafish larvae mortality at concentrations of approximately 30, 5, 4.5, and 18 mg/mL, respectively. With the increasing doses of Fuzi decoction, the mortality rates gradually increased to 100%. Then the LD_50_ value of Fuzi decoction from four producing areas were 29.62, 4.887, 18.42, and 4.223 mg/mL, respectively, calculated by a fitting professional curve. However, we could not accurately calculate the LD_50_ of Jiangyou-Fuzi, because the dose range between the initial death of zebrafish and the all death of zebrafish caused by Jiangyou-Fuzi decoction was narrow in this experiment. Considering the mortality of zebrafish larvae after administration of Fuzi decoction with different concentrations and regions, 2 mg/mL Jiangyou-Fuzi was used as a suitable exposure dose in subsequent experiments, and 3 mg/mL was used as the dosage concentration in the production areas of Anguo, Butuo, Chenggu, and Ludian.

### 3.3 Fuzi decoctions from different origins induced morphological and neurobehavioral changes in zebrafish

**FIGURE 4:**
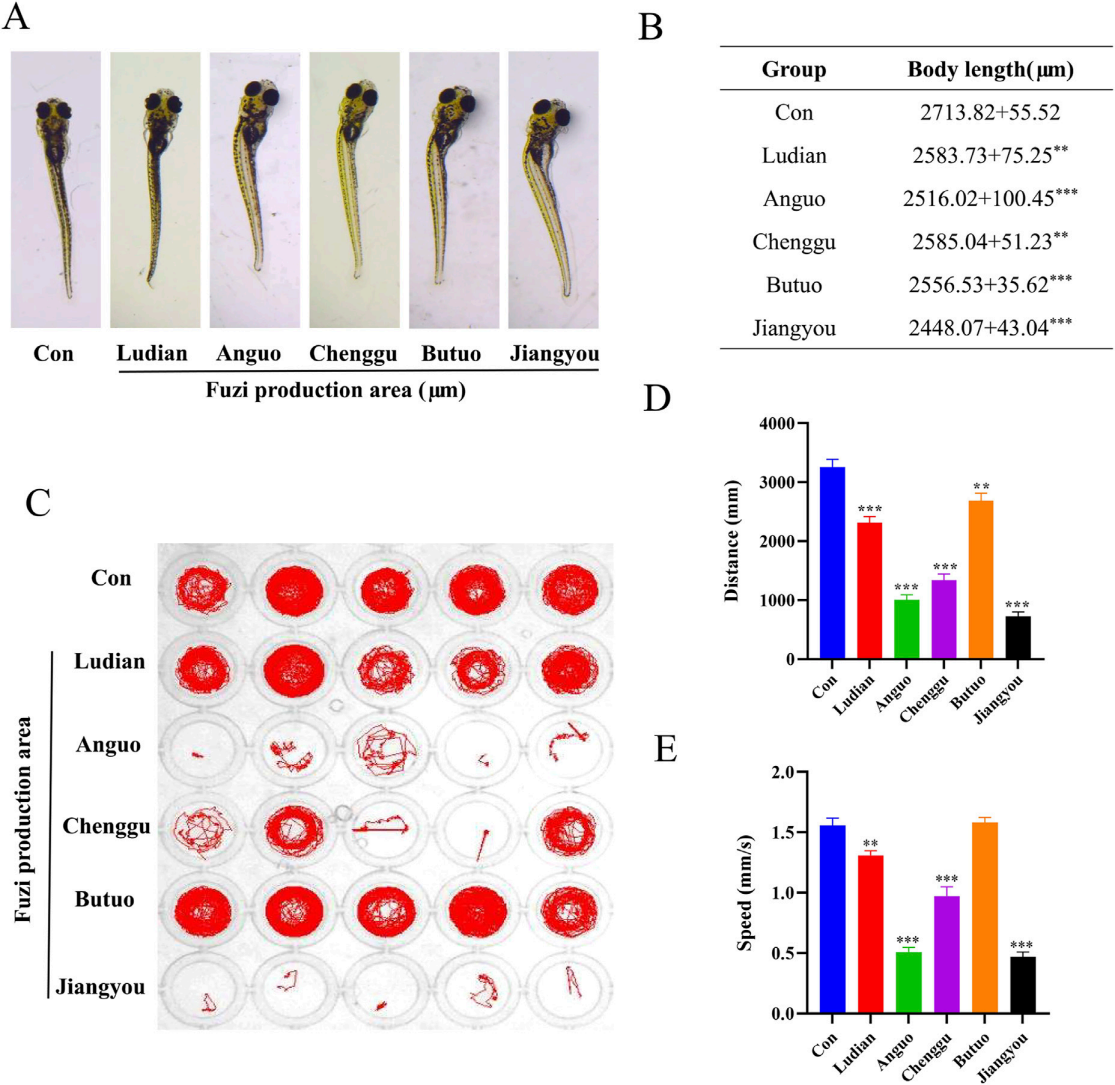
Morphological and swimming behavior changes of zebrafish larvae after Fuzi decoctions exposure. **(A)** Effects of exposure to Fuzi decoction on morphological parameters of zebrafish larvae. Images were taken under a stereomicroscope (32×), n = 6. **(B)** Body length (μm). **(C–E)** Effects of exposure to Fuzi decoction on neurobehavioral of zebrafish larvae, n = 21. **(D)** Distance traveled (mm). **(E)** Movement speed (mm/s). The values are expressed as mean ± SEM, **p < 0.01, ***p < 0.001 vs. the control group.

The teratogenic effects of Fuzi decoction from different regions on the morphology of larvae were investigated 24 h after administration, as Fuzi may affect the growth, development, and behavioral performance of zebrafish (Stewart et al., 2014). Compared with the control groups, lighter pigmentation, body curvature, and shorter body length in the Fuzi groups were observed (Figures 4A, B). Compared with the Fuzi treatment groups from other producing areas, the zebrafish larvae in the Chenggu-Fuzi treatment group had the least pigmentation, while the zebrafish larvae in the Jiangyou-Fuzi treatment group had the largest body curvature and the shortest body length. Moreover, we examined the locomotion and orientation of larvae in Fuzi decoction treatment groups from five areas to identify behavior patterns. We found that treatment with different regions of Fuzi decoction significantly decreased the distance traveled and movement speed of larvae (Figures 4C–E). Compared with the control group, there was no significant difference in the voluntary movement trajectory of the larvae, but the moving distance and speed were slightly decreased after exposure to Ludian- or Butuo-Fuzi decoction. After exposure to Anguo- or Chenggu Fuzi decoction, the autonomous movement trajectory of zebrafish was sparse and disorderly, and the moving distance and speed also decreased significantly. In the jiangyou-Fuzi decoction treatment group, the larvae of zebrafish lost the ability or willingness to move, and the distance and speed of movement were significantly lower than those of the Anguo-group or Chenggu-Fuzi group. These results indicated that the neurotoxicity of Jiangyou-Fuzi decoction was the most significant. Moreover, the larvae in all Fuzi treatment groups gradually showed unbalanced swimming, upside down of head, tail violent tremor, intermittent *in situ* “circular” violent rotation, and other abnormal behaviors, compared with those in the control groups. Therefore, we speculated that the decoction of Fuzi from different producing areas may cause the damage of motor nerves and functional loss of motor organs in zebrafish larvae to different degrees, and the neurotoxicity of Jiangyou- and Anguo-Fuzi are the most obvious.

### 3.4 Fuzi decoctions from different origins induced lipid peroxidation damage in zebrafish

**FIGURE 5:**
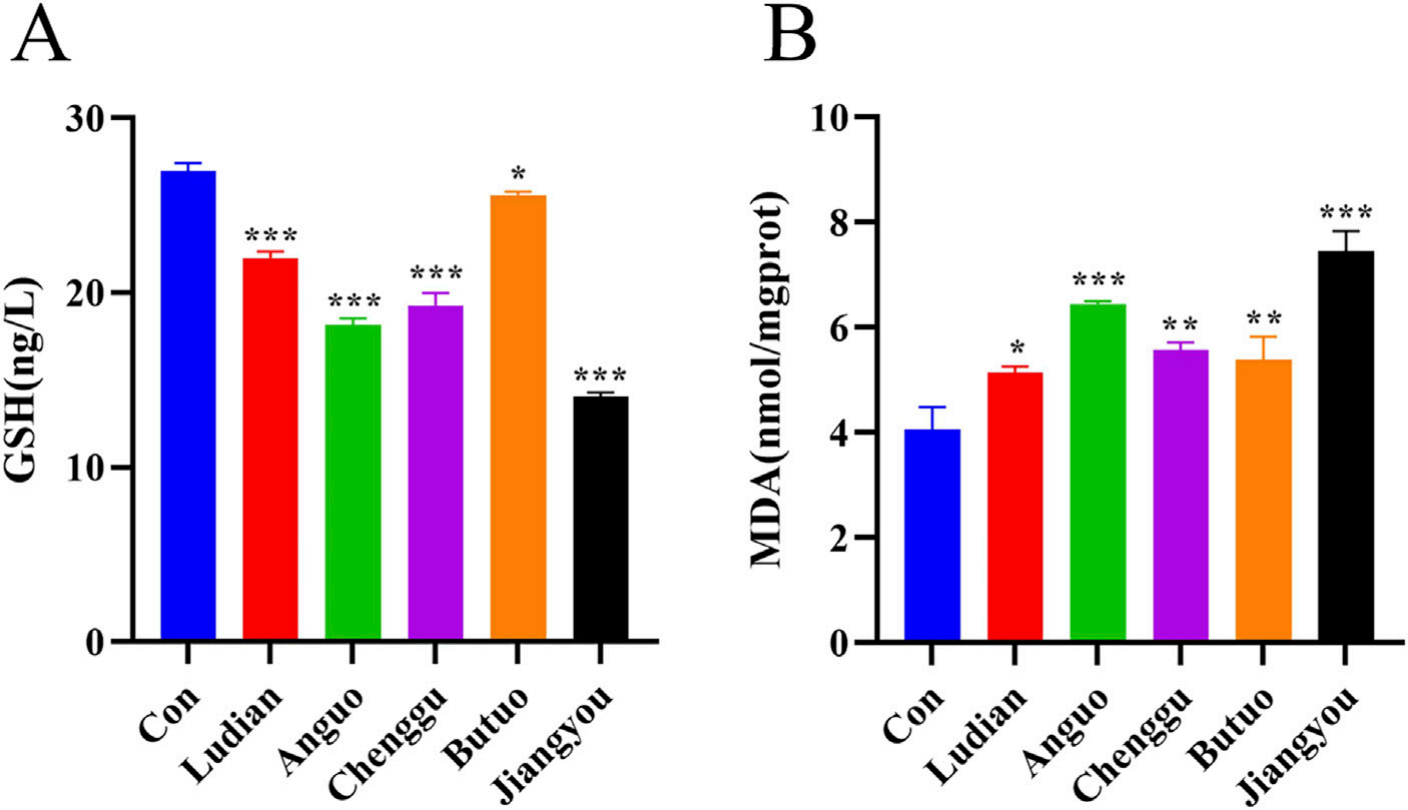
Fuzi decoctions induced lipid peroxidation in the brain of zebrafish larvae. Zebrafish at 6 dpf were treated with different regions of Fuzi decoction or fish-raising water as the solvent control for 24 h. The concentration of Jiangyou-Fuzi decoction was 2 mg/mL, and the concentration of Fuzi decoction from other regions was 3 mg/mL. **(A, B)** Levels of MDA and GSH in zebrafish larvae treated with Fuzi decoction were measured. The values are expressed as mean ± SEM, n = 25. *p < 0.05, **p < 0.01, ***p < 0.001 vs. the control group.

Lipid peroxidation is a free radical amplification process yielding a great variety of bioactive products (Schaur et al., 2015) MDA and GSH contents are important parameters reflecting the potential antioxidant capacity of the body (Sharma et al., 2016). In this study, the antioxidant and lipid peroxidation contents of larvae treated with Fuzi decoction from different regions were determined. All groups of Fuzi decoction led to a significant increase in MDA content (Figure 5A), but a remarkable decrease in GSH content (Figure 5B). Compared with Ludian-, Anguo-, Chenggu- and Butuo-Fuzi decoction treatment groups, the content of MDA in zebrafish larvae treated with Jiangyou-Fuzi was the most significantly increased. There was no significant difference in MDA in the larvae treated with Chenggu- and Butuo-Fuzi decoction for 24 h compared with Ludian-Fuzi group, but it was notably increased in the Anguo- and Jiangyou-Fuzi groups. Similar to the result of MDA, the GSH level was also the most decreased in the zebrafish larvae after Jiangyou-Fuzi decoction treatment, followed by Anguo-, Chenggu-, Ludian-, and Buto-Fuzi decoction groups. Taken together, these results confirmed that Fuzi cultivated in diverse geographical regions induced oxidative damage in the brain of zebrafish larvae to differing extents, with the Jiangyou-Fuzi potentially exhibiting the highest toxicity.

### 3.5 Fuzi decoctions from different origins affected neurotransmitter release in zebrafish

**FIGURE 6:**
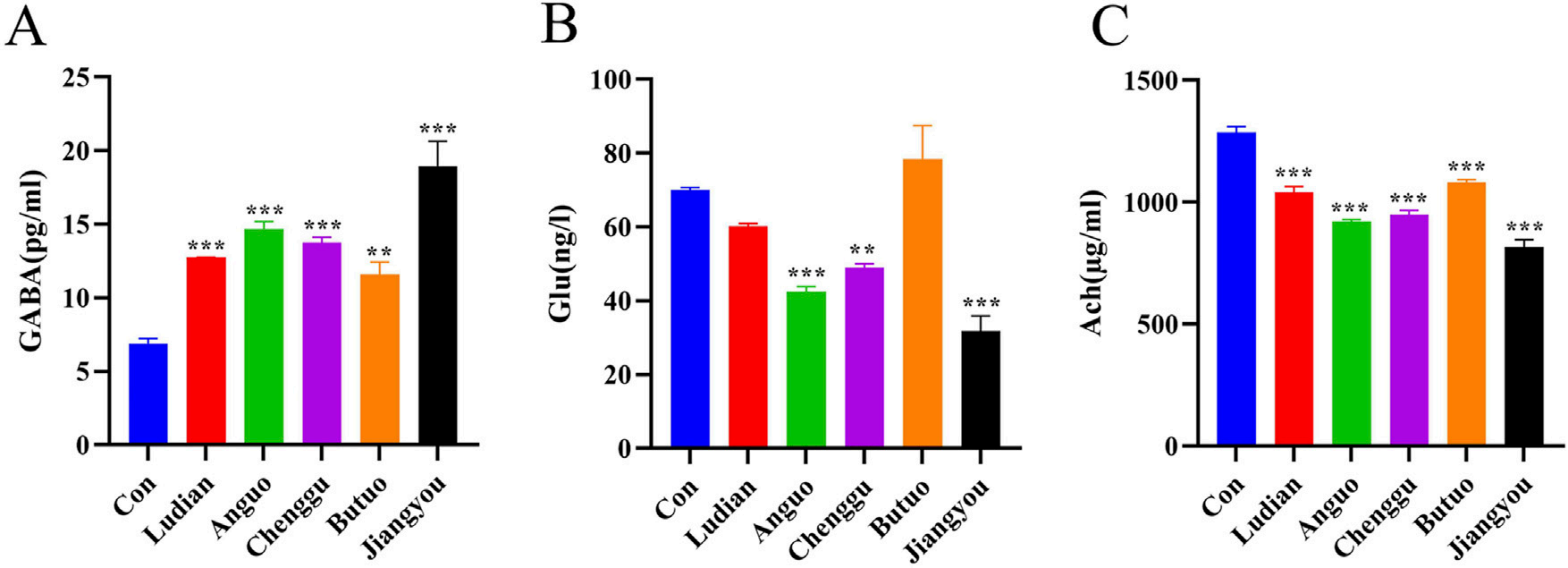
Fuzi decoctions affected the levels of neurotransmitters in zebrafish larvae. Zebrafish at 6 dpf were treated with different regions of Fuzi decoction or fish-raising water as the solvent control for 24 h. The concentration of Jiangyou-Fuzi decoction was 2 mg/mL, and the concentration of Fuzi decoction from other regions was 3 mg/mL. **(A–C)** Levels of GABA, Glu, and Ach in zebrafish larvae treated with Fuzi decoction were measured. The values are expressed as mean ± SEM, n = 25. **p < 0.01, ***p < 0.001 vs. the control group.

Neurotransmitters, which act as “messengers” in synaptic transmission of the nervous system, are capable of transmitting signals of excitation or inhibition (Pan and Peng, 2017). To explore the relationship between Fuzi decoction from various regions induced behavioral changes and neurotransmitters, the contents of GABA, Glu, and Ach in zebrafish larvae were analyzed by ELISA. Compared with the control group, there was an increase in the GABA content following exposure to Fuzi decoction from each region (Figure 6A). Notably, the highest increase of GABA was observed in zebrafish treated with Jiangyou-Fuzi decoction, followed by Anguo-, Chenggu-, Ludian- and Buto-Fuzi decoction treatment groups. Glu serves as a primary excitatory transmitter in the central nervous system (Martami and Holton, 2023). In this study, the content of Glu showed a significant decrease after treatment with Fuzi decoction from Ludian, Anguo, Chenggu, and Jiangyou regions compared with the control group (Figure 6B). However, the Glu content in the brain of the zebrafish larvae treated with Butuo-Fuzi decoction was slightly increased. Furthermore, the level of Ach in larvae was also examined (Figure 6C). When compared to the control group, the Ach levels in the brain of zebrafish larvae treated with Fuzi decoction from five regions showed a clear decrease. However, the Ach level also showed a slight increase when treated with Butuo-Fuzi decoction, compared to the group treated with Ludian-Fuzi decoction. These results suggested that Jiangyou-Fuzi decoction causes the most significant changes in neurotransmitters in the zebrafish brain, which is consistent with the neurobehavioral changes observed in zebrafish larvae in this study.

### 3.6 Fuzi decoctions from different geographical origins affected multiple nervous system pathways

**FIGURE 7:**
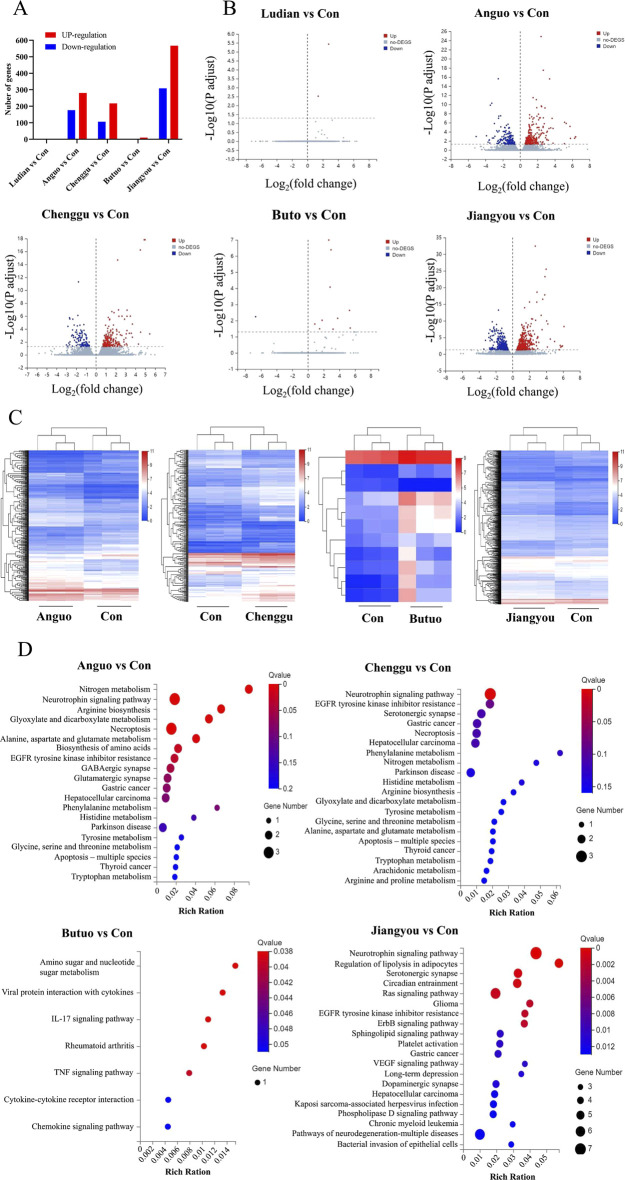
RNA-seq analysis of the DEGs between Fuzi decoctions from different regions and the control group. **(A)** The number of DEGs in five regions of Fuzi decoction treatment groups compared to the Control group. **(B)** The volcano plot of DEGs in Five regions Fuzi decoction treatment groups. Red dots are upregulated genes, blue ones are downregulated genes, and gray ones are no-difference genes. **(C)** Heatmaps show the expression patterns of DEGs in Anguo-, Chenggu-, Jiangyou-, and Butuo-Fuzi decoction exposure groups. Red rectangles are upregulated genes and blue ones are downregulated genes. The deep color means a significant difference. **(D)** Bubble charts show KEGG pathway enrichment analysis of DEGs in the zebrafish nervous system after Fuzi exposure from different regions. The color in red means a low Q-value and the area of bubbles means the gene count. n = 3.

RNA-seq-based profile analysis was conducted to elucidate the molecular mechanisms underlying the neurotoxicity induced by Fuzi from different regions in zebrafish. A DESeq analysis was employed to identify the significant DEGs with a cutoff |Log2FC| of >1 and *p* of <0.05. Then, 2 (2 upregulated genes), 456 (76 downregulated and 280 upregulated genes), 323 (106 downregulated and 217 upregulated genes), 11 (1 downregulated and 10 upregulated genes), and 875 (308 downregulated and 567 downregulated genes) DEGs were obtained in comparisons between Ludian-, Anguo-, Chenggu-, Butuo-, and Jiangyou-Fuzi vs. Control groups, respectively (Figures 7A, B). The expression profile of genes in each DEG set (Anguo-, Chenggu-, Jiangyou-, and Butuo-Fuzi decoction) in the four regions treatment groups are evaluated (Figure 7C). The heatmap results reveal that the expression changes in DEGs for Anguo-, Chenggu-, Jiangyou-, and Butuo-Fuzi decoction groups were not all in the same direction when compared to the Control group.

To further investigate the underlying neurotoxic effects of Fuzi decoction treatment on the DEGs identified in larvae, KEGG analysis was performed (Figure 7D). There were 180 differentially enriched KEGG pathways related to the nervous system between the Jiangyou-Fuzi exposure group and the control group, of which 35 pathways were significantly different. The top five significant differences were the Neurotrophin signaling pathway, Regulation of lipolysis in adipocytes, Serotonergic synapse, Circadian entrainment, and Ras signaling pathway. Compared with the control group, a total of 87 KEGG pathways related to the nervous system were enriched in the zebrafish exposed to Anguo-Fuzi decoction, of which 9 were significantly different. The top 5 *P*-values were Nitrogen metabolism, Neurotrophin signaling pathway, Arginine biosynthesis, Glyoxylate and dicarboxylate metabolism, and Necroptosis. KEGG pathways related to the nervous system were enriched in the zebrafish treated with Butuo-Fuzi decoction for 24 h. Among them, five pathways exhibited significant differences, namely Amino sugar and nucleotide sugar metabolism, Viral protein interaction with cytokine and cytokine receptor, IL-17 signaling pathway, Rheumatoid arthritis, and TNF signaling pathway. However, there was only 1 KEGG pathway with significant differences in the zebrafish treated with Chenggu-fuzi decoction, which was the Neurotrophin signaling pathway. In addition, the KEGG pathway was not enriched due to the slight interference of Ludian-Fuzi decoction on zebrafish genes. Overall, the results of RNAseq suggested that the KEGG pathways of Jiangyou-, Anguo- and Chenggu-Fuzi induced neurotoxicity in zebrafish are the same, but the degree of interference of Fuzi decoction from the two regions on the same pathway was different. The neurotoxicity of Buto-Fuzi is relatively minor, mainly interfering with amino acid metabolism and inducing inflammatory responses in zebrafish.

### 3.7 Validation of candidate DEGs and mRNA expressions related to the neurotoxicity of Fuzi

**FIGURE 8:**
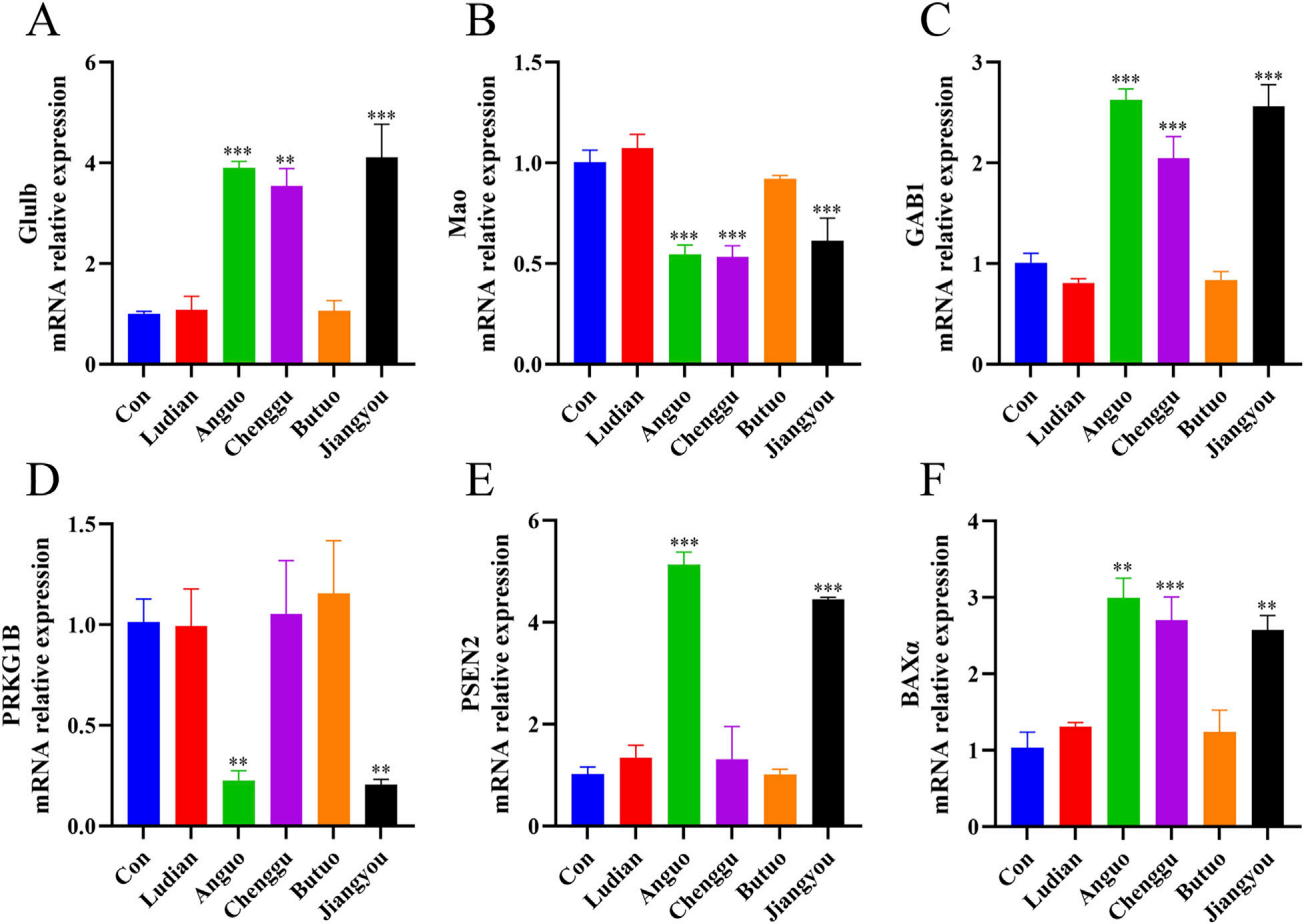
RT-qPCR verification for candidate DEGs. Zebrafish larvae were treated with/without Ludian-, Anguo-, Chenggu-, Butuo-, and Jiangyou-Fuzi decoctions for 24 h. **(A–F)** The mRNA expression of Glulb, MAO, GAB1, PRKG1B, PSEN2, and BAXα were detected by RT-qPCR to check the efficiency of RNA interference. The values are expressed as mean ± SEM, n = 25. **p < 0.01, ***p < 0.001 vs. control groups.

RT-qPCR was conducted to verify the authenticity of the results of RNA-seq. A total of 6 DEGs, including glutamate-ammonia ligase b (Glulb), monoamine oxidase (Mao), GRB2-associated binder 1 (GAB1), protein kinase cGMP-dependent 1b (PRKG1B), Presenilin 2 (PSEN2), and BCL2 associated X, apoptosis regulator α (BAXα), were screened out from the nervous system in Zebrafish. The RT-qPCR experiments confirmed that the relative mRNA expressions of these six genes were in agreement with the findings obtained from the RNA sequencing results (Figure 8). All of these genes were signally changed in at least one Fuzi decoction exposure group, and in the same direction of change in the other groups, when compared with the control group. This indicated that our transcriptome sequencing results are of reference significance to a certain extent. In the RT-qPCR results, the relative expression levels of Glulb (Figure 8A), GAB1 (Figure 8B), and BAXα (Figure 8F) mRNA in the nervous system of the zebrafish treated with Fuzi decoction from Anguo, Chenggu, or Jiangyou areas were markedly higher than those in the control group. In addition, Anguo- and jiangyou-Fuzi decoction can significantly increase PSEN2 mRNA levels (Figure 8E). Moreover, expression levels of Mao and PRKG1B in zebrafish larvae were significantly reduced after Anguo-, Chenggu-, and Jiangyou-Fuzi decoction treatment. However, the expression levels of none of the 6 genes were significantly altered after Ludian- and Butuo-Fuzi decoction treatment.

## 4 Discussion

Many Chinese herbs are produced in different regions, significantly impacting their quality and efficacy. Fuzi, a commonly used toxic traditional Chinese herb, is one such example. Modern research has demonstrated that Fuzi possesses a wide range of pharmacological effects and is widely utilized in the treatment of cardiovascular diseases, rheumatism arthritis, neuropathic pain, and bronchitis (Yang et al., 2018). Jiangyou City is the sole genuine production area of Fuzi, although it is now also cultivated in Hebei, Shanxi, and Yunnan provinces (Miao et al., 2019). Previous studies have identified significant variations in the toxicity of Fuzi sourced from different regions (Jiang et al., 2023; Li et al., 2024). Despite extensive research focusing on the cardiotoxicity of Fuzi originating from diverse geographical locations, relatively little attention has been devoted to its neurotoxicity. This study employed zebrafish larvae as a model system to explore the neurological damage induced by Fuzi from various regions and to pinpoint the crucial genes and signaling pathways implicated in this process.

In this study, we investigated the neurotoxicity of Fuzi decoctions from the Jiangyou, Anguo, Chenggu, Ludian, and Butuo regions. Diester-type alkaloids, which are the key components of Fuzi and exhibit potent pharmacological activities, are also the main contributors to its toxicity (He et al., 2023). Using the MALDI-MSI method, we detected the spatial distribution of several diester-type alkaloids in Fuzi from these regions.

The results revealed that the spatial distribution and relative concentrations of diester-type alkaloids in the transverse slices of Fuzi from Jiangyou, Chenggu, Anguo, Ludian, and Butuo regions were different, potentially explaining the variations in their neurotoxicity. The distinct spatial distribution and relative intensity of the six diester-type alkaloid components across Fuzi sections from different regions likely underlie the differing neurotoxic effects observed in Fuzi decoctions.

Subsequently, we explored the neurotoxicity caused by Fuzi decoctions from different origins. We found that five regional Fuzi decoctions treatment increased the mortality rate of zebrafish and induced deformities. Previous studies have suggested that Fuzi can inhibit the behavior of larvae, consistent with the results of our experiments (Li et al., 2019). In our behavioral experiments, Jiangyou-, Anguo-, and Chenggu-Fuzi decoctions reduced the spontaneous activity of larvae. Butuo-Fuzi decoction only affected the movement speed of zebrafish. Interestingly, larvae exposed to Fuzi decoctions from five different regions exhibited varying degrees of anxiety-like behavior. These behaviors include increased locomotor activity, heightened marginal thixotropy, and irregular, repetitive actions, which intensified with longer treatment durations. Previous studies have demonstrated that Fuzi can induce epileptic-like activity in the neocortex and hippocampus of rats, as well as produce acute and long-term excitatory effects on cerebral cortex activity (Ameri et al., 1996; Voss et al., 2008). Thus, these results confirmed that Fuzi exposure can trigger neurological impairments in zebrafish, with Jiangyou-Fuzi exhibiting the most significant toxicity.

MDA content reflects the degree of tissue oxidative damage, leading to cytotoxic effects (Tsikas, 2017). In contrast, GSH offers protection against oxidative damage through its ability to scavenge free radicals (Niu et al., 2021). We discovered that Fuzi from five different regions induce different degrees of lipid oxidative damage by regulating the levels of MDA and GSH, consistent with the results of Zhang et al. (Zhang et al., 2021). At the same time, Fuzi decoctions from various regions modulate the levels of neurotransmitters such as GABA, Glu, and Ach in zebrafish. GABA and Ach have long been considered primary inhibitory neurotransmitters (Schuske et al., 2004; Kopanska et al., 2022). Glu is the excitatory amino acid neurotransmitter (Zhou et al., 2019). Banuelos et al. (2014) found that Ach, GABA, and Glu in central neurotransmitters were closely related to learning and memory ability. The swimming behavior of zebrafish was disturbed by Fuzi from different regions, suggesting a potential correlation with the effects of Fuzi on neurotransmitter expression levels.

Notably, Jiangyou-Fuzi exhibited the highest neurotoxicity, while Butuo-Fuzi showed milder neurotoxic effects in our study. As the authentic producing area of Fuzi, Jiangyou-Fuzi boasts superior efficacy compared to other regions (Zeng and Wang, 2024). It is worth noting that the healing properties of medicinal materials not only refer to their effectiveness in treating diseases but also encompass the potential side effects on target organs. Furthermore, the roles of toxicity and efficacy can vary under different pathological (disease syndrome) conditions and dosing regimens (Peng, 2011; Peng and Peng, 2022). This may be one of the reasons for the enhanced neurotoxicity of Jiangyou-Fuzi. Additionally, the efficacy and neurotoxicity of Fuzi from different regions are influenced by various factors, including germplasm, climate, soil, cultivation methods, harvesting techniques, processing, and storage conditions (He et al., 2017; Lei et al., 2021).

Moreover, the RNA-Seq data revealed that Fuzi decoctions altered the expression levels of genes such as Glub, Mao, GAB1, PRKG1B, PSEN2, and BAXα. Obviously, larvae were more sensitive when exposed to Jiangyou-, Anguo-, and Chenggu-Fuzi decoctions than to Ludian- or Butuo-Fuzi decoctions. However, the neurotoxic phenotypes exhibited by Fuzi cultivated in five different production areas were relatively similar, involving several key pathways: the Neurotrophin signaling pathway, amino acid metabolism, the ErbB signaling pathway, the cGMP-PKG signaling pathway, and the p53 signaling pathway. The KEGG pathway analysis indicated that exposure to Fuzi decoctions significantly impacted the Neurotrophin signaling pathway, leading to the dysregulation of genes such as GAB1, PSEN2, and BAXα. GAB1 is a docking protein pivotal in linking multiple stimuli to various intracellular signaling pathways (Perez-Baena et al., 2023). The study by Zhou et al. (2020) found that GAB1 is a critical downstream effector in PDGF signaling during the differentiation of oligodendrocyte precursor cells. It regulates central nervous system myelination by modulating the activity of GSK3β and β-catenin. GAB1 also plays a role in regulating the ErbB signaling pathway, which is closely associated with the development and neuroinflammatory response of the nervous system (Mei and Nave, 2014; Kataria et al., 2019; Chen et al., 2024). PSEN2 can regulate physiological processes such as nerve cell proliferation, synapse formation, and plasticity. It is one of three proteins whose mutations can lead to early-onset familial Alzheimer’s disease (Fedeli et al., 2019). The BAXα gene plays a crucial role in cellular apoptosis, the Neurotrophin signaling pathway, the p53 signaling pathway, and pathways related to neurodegeneration (Spitz and Gavathiotis, 2022). Thus, we speculated that Fuzi can induce neurotoxicity by regulating the p53 signaling pathway, the Neurotrophin signaling pathway, and the ErbB signaling pathway by regulating the expressions of PSEN2 and BAXα. In the present study, the expression levels of GAB1 and BAXα were significantly elevated in the groups treated with decoctions of Jiangyou-, Chenggu-, and Angguo-Fuzi. Additionally, PSEN2 expression was notably increased in the Jiangyou and Anguo treatment groups, in line with the findings from RT-qPCR analysis.

KEGG analysis also showed that Fuzi regulates Glu metabolism in the nervous system by regulating the expression of genes Glulb, which is involved in the activity of glutamate-ammonia ligase and the biosynthesis of glutamine (Dhanasiri et al., 2012; Bao et al., 2013). The balance between glutamate (the precursor to GABA) and GABA (a widely present inhibitory neurotransmitter in the brain) is crucial for the proper functioning of the nervous system (Zahid et al., 2023). Consistent with RNA-seq results, Jiangyou-, Anguo-, and Chenggu-Fuzi significantly induced the expression of the Glub gene. Mao is a mammalian flavoenzyme, which catalyzes the oxidative deamination of some neurotransmitters. The concentration of biochemical neurotransmitter alterations in the brain, influenced by Mao, is directly linked to several neurological disorders, including Alzheimer’s disease and Parkinson’s disease (Manzoor and Hoda, 2020). PRKG1b can activate ATP binding activity and the cGMP-PKG signaling pathway, thereby regulating energy metabolism in the nervous system and the transmission of neurotransmitters across synapses (Li J. et al., 2018; Yang et al., 2020). Similarly, the RT-qPCR data of PRKG1b and Mao were consistent with the RNA-Seq results.

It is noteworthy that the present study, constrained by its limited scope, still has several shortcomings. Specifically, although we observed that Fuzi sourced from diverse regions disrupted the ErbB signaling pathway, the Neurotrophin signaling pathway, as well as neurotransmitter synthesis and secretion, the precise underlying effects and the extent of Fuzi’s influence on these signaling mechanisms remain largely unknown.

## 5 Conclusion

In summary, the MALDI-MSI method has achieved a breakthrough by successfully imaging aconitine, mesaconitine, hypoaconitine, benzoylaconitine, benzoylmesaconitine, and benzoylhypoaconitine in Fuzi and its various micro-regions, including the phloem, xylem, medulla, and cork, for the first time. Fuzi decoctions from different producing areas exhibited varying degrees of damage to the morphology, neurobehavior, and oxidative status of zebrafish larvae. Notably, Jiangyou-Fuzi decoction demonstrated the highest neurotoxicity, significantly impacting genes closely related to the nervous system, such as Glulb, Mao, GAB1, PRKG1B, PSEN2, and BAXα. The toxicity levels of Anguo-, Chenggu-, Ludian-, and Butuo-Fuzi followed in descending order. These findings provide a novel perspective and insights into the mechanisms underlying the neurotoxicity of Fuzi and the differences among Fuzi from various producing areas.

## Data Availability

The data presented in the study are deposited in the SRA repository, accession number PRJNA1194324.

## Abbreviations

Ach, Acetylcholine; Glu, Glutamic acid; BAXα, BCL2 associated X, apoptosis regulator α; DEGs, Differential expression genes; DHB, Dihydroxybenzoic acid; GAB1, GRB2-associated binder 1; GABA, γ-aminobutyric acid; Glulb, glutamate-ammonia ligase b; GSH, Glutathione; ITO, Indium-tin oxide; KEGG, Kyoto Encyclopedia of Genes and Genomes; LD50, Median lethal dose; MALDI-MSI, Matrix-assisted laser desorption/ionization mass spectrometry imaging; Mao, Monoamine oxidase; MDA, Malondialdehyde; PRKG1B, Protein kinase cGMP-dependent 1b; PSEN2, Presenilin 2; RNA-seq, Global transcriptome sequencing; TCM, Traditional Chinese medicine.
